# Exosome-Based Vaccines: History, Current State, and Clinical Trials

**DOI:** 10.3389/fimmu.2021.711565

**Published:** 2021-07-14

**Authors:** Patrick Santos, Fausto Almeida

**Affiliations:** Department of Biochemistry and Immunology, Ribeirão Preto Medical School, University of São Paulo, Ribeirão Preto, Brazil

**Keywords:** extracellular vesicles (EV), immunization, infectious diseases, cancer, exosomes

## Abstract

Extracellular vesicles (EVs) are released by most cell types as part of an intracellular communication system in crucial processes such as inflammation, cell proliferation, and immune response. However, EVs have also been implicated in the pathogenesis of several diseases, such as cancer and numerous infectious diseases. An important feature of EVs is their ability to deliver a wide range of molecules to nearby targets or over long distances, which allows the mediation of different biological functions. This delivery mechanism can be utilized for the development of therapeutic strategies, such as vaccination. Here, we have highlighted several studies from a historical perspective, with respect to current investigations on EV-based vaccines. For example, vaccines based on exosomes derived from dendritic cells proved to be simpler in terms of management and cost-effectiveness than dendritic cell vaccines. Recent evidence suggests that EVs derived from cancer cells can be leveraged for therapeutics to induce strong anti-tumor immune responses. Moreover, EV-based vaccines have shown exciting and promising results against different types of infectious diseases. We have also summarized the results obtained from completed clinical trials conducted on the usage of exosome-based vaccines in the treatment of cancer, and more recently, coronavirus disease.

## Exosome Functions: Biogenesis and Cargo

Extracellular vesicles (EVs) are a group of biological, nano-sized, bilayered membrane vesicles produced by almost all cells. EVs can be found naturally in body fluids, such as blood, saliva, and breast milk (1–4). Classically, EVs are classified by size, molecular cargo, and the biogenesis pathway (5). However, there was a debate in literature regarding the definition of EVs due to inconsistencies in EV purification and characterization (6, 7). Fortunately, significant progress has been achieved regarding the establishment of criteria for a standardized nomenclature of EVs, and minimal requirements are set for experimental controls during EV separation, concentration and, characterization endorsed by the International Society of Extracellular Vesicles (ISEV) (8). In terms of biogenesis, EVs can be broadly divided into two dominant classes, namely exosomes and microvesicles (MVs). Exosomes are 30-150 nm EVs that initially demonstrate formation as intraluminal vesicles inside multivesicular bodies (MVBs) and are released after fusion of MVBs with the plasma membrane (3, 6, 9, 10). Microvesicles are formed by the outward budding of the plasma membrane, a process regulated by the translocation of phospholipids (9, 11). However, according to ISEV, there is no consensus on specific markers of each EV subtype, therefore assigning an EV to a specific biogenesis remains a challenging process (8).

In the extracellular space, exosomes can undergo fusion with the plasma membrane of recipient cells and deliver their packaged cargo into the cytosol. Exosomes are highly heterogeneous vehicles that can transport a wide variety of molecules, including lipids, proteins, and nucleic acids, such as mRNAs and miRNAs. The transport of these molecules can occur within the exosome itself or *via* attachment with the surface of recipient molecules, as evidenced in the case of major histocompatibility complex (MHC) molecules (12). Healthy cells release exosomes under normal physiological conditions that play a role in several cellular processes, for example, intercellular communication by facilitating the carriage and delivery of multiple molecules that can modulate crucial processes, such as growth, differentiation, and stress response (13, 14). Thus, considerable research attention is focused on the biology of EVs. However, according to Edgar (15), emerging interest in exosome biology is attributable to the association of exosomes with disease development. Indeed, infectious, inflammatory, and neurodegenerative diseases, as well as cancer, exhibit specific biomarkers that are carried by their respective exosomes (16–18).

## History of Exosome-Based Vaccines

EV release was initially thought to be a random process; however, in 1983, two independent studies using different animal models discovered that reticulocytes released transferrin receptors inside EVs (19, 20). Barz et al. demonstrated that different lymphoma variants could produce EVs with distinct profiles of proteins and lipids that could be associated with tumor immune escape and cancer invasion (21). A year later, Schirrmacher and Barz observed that tumor-derived exosomes (TDEs) displayed antigens similar to their corresponding tumor cells (22). The same study was the first to show the anti-tumor effects of exosomes on cytotoxic lymphocytes (CTLs). In 1987, Johnstone et al. coined the term exosomes as a reference for EVs carrying transferrin receptors (23). Raposo et al. demonstrated the role of exosomes in antigen presentation by revealing MHC class II molecules in exosomes derived from B lymphocytes, which induced specific MHC class II T cell responses (24). These findings reveal that exosomes can be exploited as biomarkers and can be used in immunotherapeutic strategies for vaccine development.

The concept of a cancer vaccine is not new; it dates back to the early 70s. However, the feasibility of a vaccine against cancer is challenged by several issues, such as transplant rejection (25, 26). Tumor peptides have generated promising results and have shown potential applicability as a cancer therapeutic agent; however, peptide-based vaccines exhibit poor immunogenicity (27–29). In 1998, Zitvogel et al. (30) published a study in which they found that DEXs (exosomes derived from dendritic cells) express functional MHC class I and II molecules. They observed that tumor peptide-pulsed dendritic cells (DCs) released DEXs presenting tumor antigens on the membrane, which induced *in vivo* CTL priming and consequent tumor growth suppression. This study was the first to support the development of a novel cell-free vaccine using exosomes, representing a milestone in exosome-based vaccine research.

In the new millennium, Wolfers and colleagues have reported that TDEs represent a source of T-cell cross-priming which is realized *via* transfer of antigens to DCs, and this induces CTL anti-tumor responses *in vitro* and *in vivo* (31). During *in vitro* stimulations, TDEs were more effective in eliciting protection against autologous tumors than other cancer immunization strategies, such as irradiated tumor cells, apoptotic bodies, or tumor lysates (31, 32). In 2004, the Zitvogel group published two articles that comprehensively described the transfer of MHC class I molecules from DEXs to naïve DCs for efficient CTL activation, and the role of toll-like receptors in combination with DEXs in triggering an MHC-restricted response in CD8^+^ T cells using *in vitro* stimulations and HLA-A2 transgenic mice (33, 34). In the same year, exosomes released by plasmacytoma cells were successfully used as a cancer vaccine; in this case, plasmacytoma exosomes conferred protection to the animals through reduction in tumor growth by 80% after a single vaccination (35). The use of exosome-based vaccines has since spread to different research areas outside cancer therapy. Exosomes derived from DCs previously co-cultivated with *Toxoplasma gondii* generated a strong and specific immune response to induced acute and chronic toxoplasmosis (36). Further investigation using *Toxoplasma gondii* has been detailed in section 5. Exosomes derived from an antigenic extract of *Salmonella enteritidis* strain were isolated and cultivated with serum samples obtained from naturally infected and healthy chickens (37). After completion of the exosome treatment, surface structures from *Salmonella*, such as flagellin and porins, were found to be immunogenic in serum samples collected from infected chickens but did not exhibit immunogenicity in healthy ones. These results represented a concrete evidence highlighting that *Salmonella*-derived exosomes could be used in the preparation of vaccines (37). Recently, a vaccine was designed by using a plasmid to generate *Salmonella* exosomes containing highly immunogenic membrane antigens and it showed satisfactory immune responses against several *Salmonella* strains (38).

**Figure 1:**
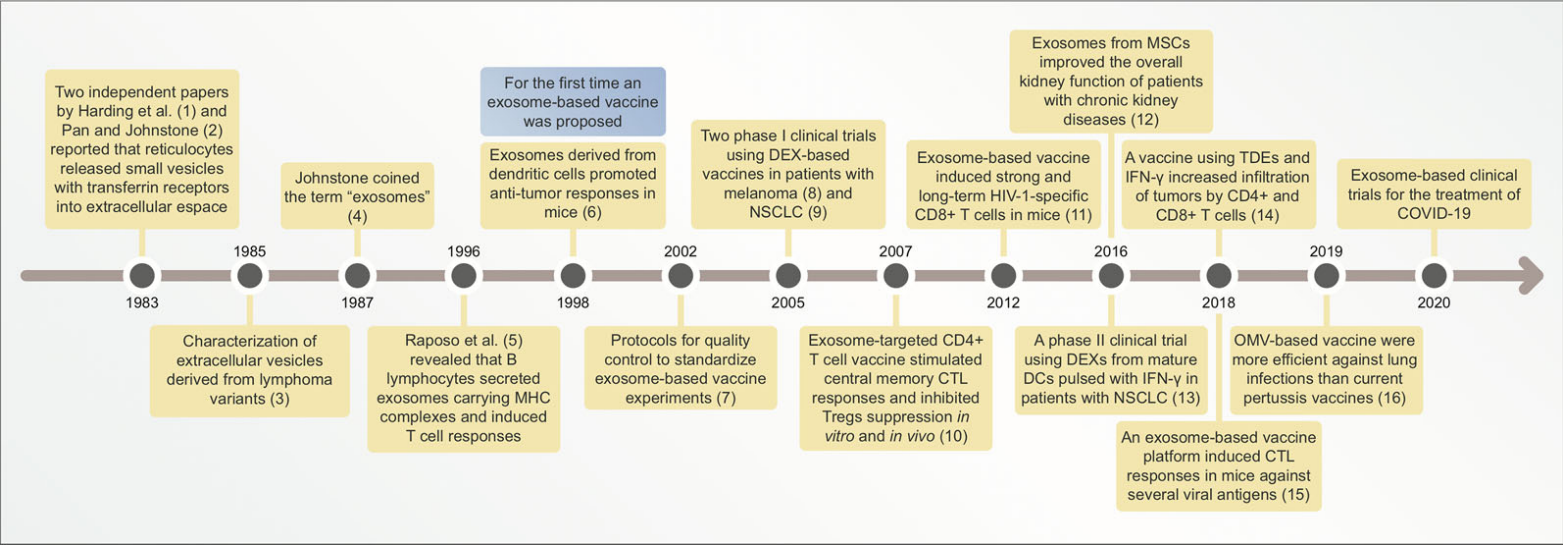
Timeline illustrating main discoveries related to exosome-based vaccines. CTL, cytotoxic lymphocyte; DEXs, exosomes derived from dendritic cells (DCs); NSCLC, non-small cell lung cancer; MSCs, mesenchymal stem cells; TDEs, tumor-derived exosomes; OMV, outer membrane vesicle; COVID-19, coronavirus disease-19.

From this point onward, exosome research increased due to the development of more sophisticated techniques, such as exosome engineering for drug delivery systems and artificial antigen presentation models (**Figure 1**). In the mid-2000s, the first results from clinical trials on exosome-based vaccines were reported (39–43). Clinical trials using exosome-based vaccines have been detailed in section 6. Currently, several exosome-based vaccine candidates are under development for diseases such as cancer, AIDS, hepatitis B, and other infectious diseases (44–48). The vaccines have been discussed in further detail in the subsequent sections.

## Exosome-Based Vaccines as a Cancer Therapeutic Strategy

Tumor cells can evade immune surveillance through several regulatory mechanisms, such as reduced immune recognition or the establishment of an immunosuppressive tumor microenvironment (49). In this scenario, cancer cells can undergo proliferation and facilitate the recruitment of immune and stromal cells to favor tumor progression, which can lead to metastasis (50). Cancer immunotherapy has emerged as a clinical strategy for controlling the immune system and for reactivating anti-tumor immune responses (51). Immunotherapy approaches include targeting of immune tolerance *via* co-inhibitory checkpoints, adoptive T-cell therapy, and cancer vaccination (52).

Cancer vaccines differ from traditionally engineered vaccine for infectious diseases in the intervention approach. Traditional vaccines are preventive, on the other hand, cancer vaccines are focused on the therapeutic aspect. However, there are prophylactic interventions to reduce cancer incidence, morbidity, and mortality for virus-related cancers, such as hepatitis B (HBV) and human papilloma virus (HPV) (53). Therapeutic cancer vaccines can target a wide variety of antigens expressed by cancer cells, including antigens that are exclusively expressed in cancer cells, also known as tumor-specific antigens (TSAs), for example, mutated P53 and RAS. Cancer vaccines can also target antigens that have low levels in normal but highly expressed in tumor cells, the tumor-associated antigens (TAAs), such as MAGE-1, HER2, and HPV (54–56). There are also different platforms available, such as peptide-based, DNA-based, protein-based, viral-based, whole cancer cells, recombinant factors, and pulsed DCs (53, 56–58). Currently, only three cancer vaccines are approved for clinical use by FDA to treat early-stage bladder cancer (TheraCys^®^), metastatic castration-resistant prostate cancer (PROVENGE), and metastatic melanoma (IMLYGIC^®^). These vaccines have produced slightly improved overall survival of patients with early-stage disease (58). For patients with advanced or metastatic tumors, cancer vaccines are likely to have a therapeutic role in a combination therapy approach (59).

Despite suboptimal results, recent cancer vaccine interventions are clinically promising and have shown potential applicability, especially with respect to overall patient survival (60). According to Melief et al., a robust cancer vaccine design must enable the induction of potent effector CD4^+^ and CD8^+^ T-cell responses (60). Target antigen selection is challenging; selection is based on overexpressed antigens in tumors relative to normal tissue (61). Owing to the immunosuppressive tumor microenvironment, cancer vaccines should be administered in combination with adjuvants to overcome immunosuppression (62). Adjuvants are key components of several successful vaccines that boost the vaccine’s immune response, quality, and efficacy (63). An interesting strategy for vaccines based on TAAs is the use of a combination of adjuvants and immunomodulatory antibodies (62). Exosomes exhibit features for application as adjuvant carriers, such as optimal size, biocompatibility, stability in systemic circulation, and target-specific delivery (64). Recently, an exosome-based adjuvant delivery system was developed using genetically modified murine melanoma B16BL6 cells, in which the exosomes derived from these cells containing CpG DNA were injected three times with a 3-days intervals and successfully induced immunostimulatory signals in mice 7 days after the last immunization (65). These results shed light on the novel use of exosomes as adjuvant carriers for future cancer vaccine development. Adjuvant strategies to increase cancer vaccine efficacy have been thoroughly reviewed by Bowen et al. (62).

**Figure 2:**
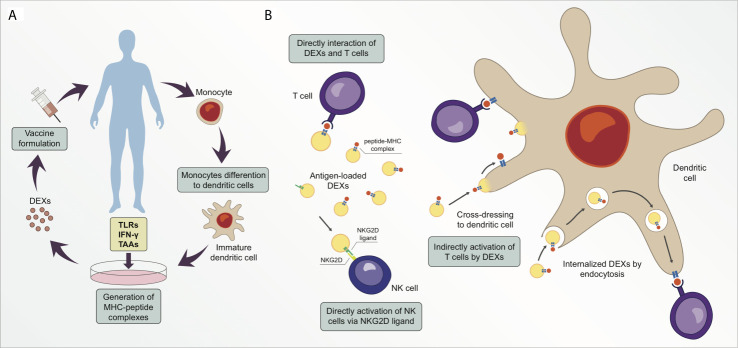
Exosomes derived from dendritic cells (DEXs) are potential targets for cancer therapeutic strategy. **(A)** Simplified illustration of a personalized vaccine using DEXs. **(B)** DEXs can directly catalyze the transfer of peptide-MHC complexes from their membrane surface to T cell membrane surface (cross-dressing). Moreover, DEXs can stimulate T cell responses in an indirect manner *via* cross-dressing with dendritic cells or *via* exosome uptake and processing, following the peptide-MHC complex presentation to T cells. DEXs can also induce activation and proliferation of NK cells by establishing interaction of the NKG2D ligand on DEXs with NKG2D receptors on the NK cell membrane.

To design a successful cancer vaccine, researchers must also consider administration routes and optimal delivery vehicles. DC injection is a common delivery system that triggers initiation and controls the direction of antigen-specific immune responses (64). However, DC-based immunotherapy has shown inconclusive results in clinical trials. Moreover, DC vaccines are an expensive therapeutic strategy for implementation in large populations, and they are difficult to ensure standardized production and lose efficacy over long periods of storage (49, 66). DEXs have emerged as a viable option for cancer vaccination because they possess higher stability for a longer period than DCs because of their lipid composition. DEXs also possess more peptide-MHC I and -MHC II complexes than DCs, thereby rendering the use of DEXs a less time- and space-consuming strategy (**Figure 2A**) (66–68). Additionally, DEXs are more resistant to immunosuppressive mechanisms in the tumor microenvironment than DCs (69). Exosomes are reportedly more capable of inducing immunocompetence in DCs than in microvesicles. An *in vivo* comparison of immunostimulatory potential between microvesicles and exosomes derived from ovalbumin (OVA)-pulsed DCs showed that only exosomes induced antigen-specific CD8^+^ T cells and increased the proportion of germinal center B cells. Exosomes were also superior in terms of OVA levels, while microvesicle-associated OVA was barely detectable; however, microvesicles and exosomes both induced higher OVA-specific IgG production relative to controls (70).

Several studies revealed that DEXs can activate CD4^+^ and CD8^+^ T cells, indicating the ability of DEXs successfully carry antigen-MHCI/II complexes *in vivo* and *in vitro* (34, 71–73). Once activated, CD8^+^ T cells can become memory T cells. Wang et al. using a melanoma mice model, induced the CD8^+^ T cells differentiation to CTLs *via* DEXs from mature DCs. Three months after the immunization, the immunized mice group was boosted with DEXs and the number of CD8+ T cells expressing antigen-specific T cell receptor (TCR) was expanded six- to seven-fold in immunized mice. Another experiment in this study was to challenge immunized mice and control groups with melanoma cells three months after immunization protocol. Immunized mice were tumor free and control mice died of lung metastases. Moreover, these antigen-specific CD8+ T cells express CD44, a marker for memory T cells (74). The immunological memory induced by DEXs was also observed in CD4+ T cells of mice treated with OVA-pulsed DEXs, which induced an immune response towards to Th1 type. Interestingly, in this study, an efficient long term memory response of OVA-specific Th1 cells after a boost was dependent of prior B cell activation (75). CD4^+^ cells after uptake OVA-pulsed DEXs could stimulate efficient antigen-specific CTL responses and long-term T CD8^+^ cell memory in immunized C57BL/6 mice against OVA-transfected melanoma cells expressing OVA challenge after three months of complete immunization (76). On the other hand, DEX vaccines failed to induce antigen-specific T cell responses in clinical trials (further discussed in section 6). Preclinical results showed that DEXs released by DCs treated with interferon-γ (IFN-γ) express high levels of molecules capable to induce a strong CD8^+^ T cell activation, such as CD40, CD80, CD86, and CD54 (77). However, this enhancing DC strategy did not translate into results in a phase II clinical trial, which the peptide-specific T cell responses were not detectable (43).

Recently, a combination of cancer vaccination and checkpoint blockade strategies was designed to induce anti-tumor responses *in vitro* and *in vivo*. Exosomes released by modified anti-CTLA-4 antibody and OVA-pulsed DCs (DEXs_OVA-CTLA-4_) were enriched in MHC I/II molecules and were found to exert strong T-cell activation and proliferation *in vitro*. Vaccination with DEXs_OVA-CTLA-4_ increased the migration of CD4^+^ and CD8^+^ T cells to the tumor site and elevated the ratio of CTLs/Tregs in the microenvironment of B16 melanoma tumor model after 12 days (78). Hao et al. demonstrated that exosomes derived from OVA-pulsed DCs and their uptake by CD4+ T cells stimulated the proliferation and differentiation of central memory CTLs and inhibited Treg suppression *in vitro* using BL6 melanoma cells. Also, in this study, C57BL/6 mice immunized with OVA-pulsed DEXs showed an elevated number of OVA-specific CD8^+^ CD44^+^ T cells three months after the immunization in comparison to control group (42). Long-term functional CTL memory was observed in animals injected with OVA-pulsed DCs and was then challenged with OVA-expressing B16 melanoma cells (79). Different mechanisms of antigen presentation by DEXs have been proposed (**Figure 2B**) (80, 81). Recipient DCs may establish interaction with antigen-loaded DEXs *via* the endosomal pathway, followed by the transfer of the peptide-MHC complex to the DC surface membrane for antigen presentation to T cells (82). Furthermore, a second indirect antigen presentation mechanism called cross-dressing occurs when an acceptor DC captures DEXs by facilitating the merging of membranes and retains the peptide-MHC complex on the DC surface without processing (80, 83). The direct interaction of DEXs with T cells seems to demonstrate poor efficiency in stimulating T cell responses, therefore DEXs have less T cell stimulation potential than their parent DCs (66, 84). Some authors suggest that exosomes are not able to interact directly with effector cells, thus prior capturing and processing the exosomes by DCs is a superior pathway of priming specific T cells *via* DEXs (75, 81, 85, 86). A study using the direct interaction of DEXs with T cells showed that DEXs from mature DCs are better at stimulating T cells than DEXs from immature DCs (87). Robbins and Morelli suggest that the low ability of exosomes to stimulate T cells *in vitro* is probably due to the small size and dispersion of exosomes caused by Brownian motion (88). These authors also suggest that T cell stimulation by exosomes can be enhanced when exosomes are immobilized and at high concentration (88).

Damage-associated molecular patterns (DAMPs) are signaling molecules released by dying cells that trigger immune cells to activate defensive mechanisms (89). For example, tumor-derived DAMPs establish interaction with Toll-like receptors (TLRs), which directly lead to the activation of T cells and indirectly result in the induction of the release of inflammatory cytokines (90). Damo and colleagues developed different exosome vaccines derived from OVA and TLR ligand-pulsed bone marrow DCs (91). Their results showed that the TLR-3 ligand-DEXs vaccine (OVA + poly I:C) stimulated higher antigen-specific CD8^+^ T-cell proliferation and effector functions and increased the population of TNFα^+^CD4^+^ T cells in the lymph nodes of vaccinated mice with melanoma compared to other vaccine formulations 19 days after priming. Additionally, this group showed that purified DEXs successfully carried melanoma epitopes and induced potent anti-tumor immune responses, thereby slowing tumor progression. Recently, a DEX-based vaccine combined with microwave ablation was reported to inhibit tumor growth in hepatocellular carcinoma (HCC) mouse models compared to microwave ablation (a common therapy for HCC patients) alone, in this case, the tumor disappeared 10 days after microwave ablation in combination with DEX injection (92). Additionally, HCC features a high expression level of α-fetoprotein (AFP), which has been used as an HCC antigen for monitoring and diagnosis (93). DEXs from AFP-enriched DCs generated strong antigen-specific immune responses *in vitro* tumor suppression after 26 days in HCC mice under a vaccination regimen of a weekly injection for three weeks (71).

In addition to carrying MHC complexes on their surface, DEXs carry proteins that can stimulate cells of the innate immune system. For example, a study showed that DEXs expressing BAT3 on the surface, which is a protein responsible for engaging natural killer (NK) cell activation, induced NK cell-mediated cytokine release *in vitro* (94). DEXs induced a strong NK cell activation and stimulated the release of IFN-γ in a dose-dependent manner *via* TNF in mice (95). DEXs also express several other ligands on their surface that can mediate innate immune functions, such as TNF, FasL, and TRAIL (95). Moreover, the DEX membrane contains the activating receptor NKG2D ligand, which is responsible for the activation and proliferation of NK cells (96).

Although DCs have been pulsed with TLRs, biomarkers, and tumor antigens derived from lysates, TDE-pulsed DCs were reported to generate the most remarkable results as a potential anti-tumor vaccination. As mentioned earlier, TDEs provide a broad range of TAAs for antigen presentation. TDEs also transfer mRNAs and non-coding RNAs, such as miRNAs and long non-coding RNAs (lncRNAs) (97, 98). Recent data suggests that mRNAs packaged inside TDEs are responsible for stimulating the immune response by MHC I cross-presentation to DCs (99–101). For example, TDEs derived from CD40L/4-1BBL-expressing Mel526 melanoma cells induce potent DC activation *in vitro* (100). The interaction of 4-1BBL with its receptor 4-1BB results in the formation of a complex that induces CD8+ T cell activation and expansion (102). Interestingly, peptides derived from introns and exons of mRNAs derived from mouse melanoma cells act as tumor-associated peptides that can be delivered to DCs and result in the promotion of CD8+ T cell activation and proliferation (99). A recent study using sequencing technology showed that exosomes derived from plasma of 150 patient with cancer contained abundant levels of lncRNAs that could act as potential biomarkers for cancer diagnosis, specially 5 lncRNAs that can serve as HCC biomarkers diagnosis (103). Exploitation of lncRNAs derived from TDEs seems promising as a vaccination approach. For example, *LINC02195* is an lncRNA capable of regulating MHC I molecules during antigen processing and presentation (104). Furthermore, a signature was identified as a prognostic predictor of laryngeal cancer using the lncRNAs of TDEs (105).

A vaccine designed using TDE-loaded DCs showed superior immune response induction compared to tumor lysate-loaded DCs as evidenced by results obtained in mouse myeloid leukemia and renal cell carcinoma models (106). Recently, the same effect was observed in lung cancer, in which TDE-pulsed DCs induced a reduction in the population of regulatory T cells (Tregs) *in vitro*, while they suppressed tumor growth and increased animal survivability *in vivo* (107). DCs pulsed with TDEs derived from different types of cancers (such as leukemia, renal carcinoma, glioblastoma, and pancreatic cancer) elicit anti-tumor immune responses (108–112). DC activation and maturation can be induced by the high-mobility group nucleosome-binding protein 1 (HMGN1), a well-known Th1-polarizing alarmin (113, 114). TDEs bound to the N-terminal portion of HMGN were found to induce persistent anti-tumor immunity in orthotopic HCC mice (115).

In most studies, TDEs derived from patient sera have been found to be biocompatible and exhibit low immunogenicity. However, it is relevant that TDEs play roles in all steps of cancer progression, including metastasis and they can be immunosuppressive in certain types of cancer (115–117). The immune-suppressive potential of TDEs has been reported to inhibit the effector activity of CD4+ and CD8+ T cells and NK cells (118). Recently was demonstrated that TDEs can carry the programmed death ligand (PD-L1), which is responsible for T cell exhaustion (119). Moreover, TDEs can block the differentiation of DCs, induce apoptosis, and diminish the overall T cell responses in different types of cancer (120–122). In addition, several studies show that TDEs have potential to suppress the effects of therapeutic agents (123, 124), for example, TDEs are associated to acquired chemoresistance (125).

TDEs may also exert a dual effect, improving DC vaccine efficiency *in vitro*, while favoring tumor progression *in vivo* (117). Immunomodulatory molecules combined with TDEs may induce enhanced anti-tumor immune responses. For example, a vaccine designed with TDEs released by mouse cancer cell lines subjected to treatment with IFN-γ and interferon receptor factor-1 (IRF-1) was found to increase the number of infiltrated CD4+ and CD8+ T cells and reduce tumor size in C57BL/6J female mice transfected with Hepa 1-6 hepatoma cells or MC-38 colon carcinoma cells after 21 days of the exosome injection (46). Additionally, in a recent study reported by Shi et al., a vaccine with exosomes derived from IFN-γ-modified RM-1 prostate cancer cells under a vaccination regimen of 4 injections (on days 0, 4, 8, and 12), decreased the number of Tregs and reduced the tumor metastatic rate in C57BL male mice with lung metastasis (126). These findings indicate that pulsing DCs with a wide variety of molecules can help produce exosomes capable of generating a robust anti-tumor immune response (**Table 1**). These methods represent promising and potentially individualized TDE- and DEX-based vaccine strategies for cancer immunization.

**Table 1:** Different experimental models and design using exosomes to induce anti-tumor immune responses against several types of cancer.

Experimental model	Cancer type	Experimental design	Clinical outcome	Reference
C57BL/6 mice; Hepa1-6, 4T1, Hela, and EL4 cell lines	HCC	Intravenous injection of DCs pulsed with TDE-N1ND	Generation of long-term memory T cells and robust anti-tumor immunity	(115)
C57BL/6 and IRF3-KO mice; E0771 cell line	Breast	Cancer cells treated with topotecan	TDEs from treated cells contain immunostimulatory DNA	(127)
C57BL/6 mice; A549 and LLC cell lines	Lung	Vaccination with 3 doses of DCs pulsed with TDEs	TDEs promoted DC maturation, which increased tumor-infiltrating CD8^+^ T cells in mice	(107)
Zipras/myc-9-infected C57BL/6	Prostate	Vaccination with 4 doses of TDEs pulsed with IFN‐γ	Prolonged survival time, attenuated expression of PD-L1, reduced tumor metastasis rate	(126)
C57BL/6 and CD45.1 mice	–	Antigen transfer from DEXs released by plasmacytoid DCs to conventional DCs	Cross-priming of naïve CD8^+^ T cells	(128)
C57BL/6 and BALB/c mice; Hepa1-6, RAW264.7, LLC, and 4T1 cell lines	Lung and liver	Vaccination with a single dose of exosomes from cancer-bearing mice after photothermal therapy	Promoted infiltration of T cells into the tumor tissue	(129)
Transgenic HLA-A2/HER2 mice; 4T1 and BT474 cell lines	Breast	Vaccination with a single dose of DEXs from HuRt-specific DCs transfected with an adenoviral vector	Activation of CD8^+^ T cell cytolytic functions against breast cancer cells in *vitro* and reduced tumor growth in *vivo*	(130)
BALB/c and C57BL/6 mice; H22, B16, and CT26 cell lines	Melanoma, liver, and colon	Vaccination with 3 doses of TDEs released by different cancer cell lines	Promoted DC maturation and elicited T cell anti-tumor responses	(131)
HepG2 and K562 cell lines	HCC	Isolation of exosomes released by cancer cells treated with anti-cancer drugs	TDEs exhibited heat shock proteins in their surface that activated NK anti-tumor response	(132)
4T1	Breast	Modified TDEs with microRNAs to enhance their immune stimulation function	Modified TDEs induced DC maturation *in vitro*	(133)

## Exosome-Based Vaccines for Treatment of Viral Infectious Diseases

Similar to cancer, exosomes act as a double-edged sword because of their ability to carry and deliver molecules to target cells in infectious diseases. Exosomes play a crucial role in the pathogenesis of infection, but also trigger immune responses to confer protection against pathogens (134). This effect can be observed in the context of viral infections, where exosomes derived from infected cells can deliver viral content to surrounding cells, but can also induce antiviral immune responses (135). The “Trojan exosome” hypothesis proposed by Gould et al., describes the evolutionary similarities of viruses and exosomes with regard to their biogenesis and transmission pathways, suggesting exosomes as a potential tool for vaccination against human immunodeficiency virus (HIV) (136). The exosomal biogenesis pathway that is hijacked by HIV for viral spread can be exploited as a potential therapeutic approach (137).

Efforts are ongoing to evaluate the potential of exosome-based vaccines against HIV. Dr. Jim Xiang’s research group pioneered this research area and developed a vaccine termed as Gp120-Texo. This vaccine was designed with DEXs derived from DCs transfected with an adenoviral vector, AdV_Gp120_, which expressed the HIV-specific envelope glycoprotein Gp120 (138). Gp120-Texo induced strong and long-term HIV-specific CD8^+^ T-cell responses independent of CD4^+^ T cells and DCs in mice (44, 138). Later, the Xiang group designed a vaccine to induce a specific immune response against Gag (Gag-Texo) (139), a group of proteins responsible for HIV maturation and infection (140). Gag-Texo induced Gag-specific immunity in animal models of chronic infection, suggesting that this vaccine might induce CTL responses to attack HIV-infected cells (139). Nef is an HIV protein associated with multiple cellular functions, such as the survival of infected cells and vesicular trafficking (141). An exosome-based vaccine was engineered by incorporating a Nef mutant (Nef^mut^) into exosomes. In this case, Nef^mut^-exosomes were absorbed by DCs, which then presented the antigens, thereby eliciting CTL immune responses in mice against several viral antigens, such as those for HIV, Ebola, influenza, HBV, and hepatitis C virus (HCV) (47, 142, 143).

Even with current diagnostics and therapeutics that enable viral suppression, HBV continues to represent a major healthcare concern worldwide (144). HBV is frequently associated with the development of chronic liver diseases, such as HCC (145). Exosomes released by HBV-infected cells contain several proteins encoded by the HBV genome, as well as miRNAs that regulate gene expression in host cells (146–148). This sheds light on the potential use of exosomes to understand HBV transmission and HBV-host interactions. However, there is a lack of literature on exosome-based HBV vaccination. Few studies have investigated the potential of a general exosome-based vaccine platform for multiple viral antigens, including HBV. Additionally, a vaccine formulation designed with unmodified exosomes as adjuvants for the recombinant HBV antigen showed promising results, in which exosomes induced a Th1 immune response, thereby enhancing the levels of IFN-γ in mice (149). These studies are in the early phase, and further investigations are warranted to identify therapeutic targets for consideration as vaccine candidates against HBV using exosomes as delivery systems or adjuvants.

Influenza virus infection is another example of a healthcare concern that causes significant morbidity and mortality worldwide (150). Despite the wide variety of vaccine types available for influenza infection, studies have shown that exosomes can be used as a new platform for designing influenza vaccines, with exhibition of advantages over classical vaccines (151, 152). For example, airway exosomes released during influenza virus infection can carry host proteins with anti-influenza properties and can help trigger immune responses (153). A study using LC-MS/MS showed that exosomes derived from infected cells also carried similar proteins as those reported in the influenza virions, representing an alternative pathway for the infection of new host cells (154). Lung and serum-derived exosomes from mice infected with influenza virus exhibit high levels of miR-483-3p, and this is associated with the induction of pro-inflammatory cytokine release (155, 156). According to the authors, further studies are warranted to determine whether the transfer of miR-483-3p is involved in the activation of innate immune responses or in the inflammatory pathogenesis of influenza virus infection. Another exosome-based vaccination approach to combat the influenza virus includes EVs released by gram-negative bacteria, which are referred to as outer membrane vesicles (OMVs) (157). Several recent studies have reported that OMV-derived vaccines can induce strong immune protection against the influenza virus *in vivo* (158–161).

## Exosome-Based Immunization Strategy for Non-Viral Infectious Diseases

The release of exosomes by non-viral pathogens such as bacteria and parasites, plays an important role in pathogenesis by establishing interactions with the host immune system and by transferring resistance factors (162). However, exosomes and OMVs derived from bacteria have been reported to be potent immune modulators, rather than aiding pathogenesis (163). The potential of OMVs as immune activators has been investigated using models of different infectious diseases such as pertussis (whooping cough), which is caused by *Bordetella pertussis*, a gram-negative bacterium (164). Currently available vaccines aid the successful reduction of the morbidity and mortality caused by pertussis, but they are also associated with severe adverse effects and weak immune protection (165). According to the World Health Organization (WHO), there is no consensus regarding the antigenic composition of an optimal pertussis vaccine (https://www.who.int/biologicals/areas/vaccines/apertussis/en/). Several studies have now shown that a *B. pertussis* OMV-based vaccine can overcome this composition issue, representing an attractive vaccination model for pertussis (166–168). A recent OMV-based vaccine conferred protection to mice against lung infection more effectively than the current commercial pertussis vaccines (48). Although overshadowed by gram-negative bacteria, EVs derived from gram-positive bacteria have also recently gained attention as a potential vaccine platform for several infectious diseases. EVs released by *Staphylococcus aureus* were modified to possess no toxicity and to serve as vaccine candidates. Genetically engineered EVs showed immunogenic effects and protected mice against lethal sepsis caused by *S. aureus* (169). Additionally, EVs derived from *Streptococcus pneumoniae* incubated with murine DCs were rapidly internalized and enhanced the release of tumor necrosis factor (TNF)-α, which constitutes the inflammatory response (170).

Investigations of exosome-based vaccines for infectious diseases are not limited to viruses and bacteria. Toxoplasmosis is a globally occurring infectious disease caused by the coccidian protozoan *Toxoplasma gondii* (171). Vaccines with live and attenuated tachyzoites are available for animals; however, these vaccines are not effective and safe for humans (172). Therefore, the development of a toxoplasmosis vaccine for humans is of considerable interest for public health. However, few studies have reported the effects of DEXs derived from DCs pulsed with *T. gondii* or *Toxoplasma*-specific antigens (36, 173). In a recent study, DEXs released by DCs stimulated with *T. gondii* lysate were inoculated intranasally and ocularly in mice, which subsequently triggered humoral and mucosal immune responses against *Toxoplasma* infection (174).

Schistosomiasis is a major parasitic disease caused by *Schistosoma mansoni*, affecting a myriad individuals and causing over 280,000 deaths annually worldwide (175). Thus far, there is no vaccine available for schistosomiasis, which underscores the need for the development of vaccines against this disease. Few authors have suggested the use of exosomes as a cell-free vaccination platform against *S. mansoni* infection (176–178). Exosomes released by *S. mansoni* adult worms contain miRNAs and proteins involved in host-parasite interactions, such as invasion, nutrient acquisition, and immunomodulation (178). A study showed that *S. mansoni*-derived exosomes harbored several potential vaccine candidates, including proteins involved in multiple life cycle stages, underlining their potential utility in different stages of the parasite’s life cycle (176). These findings represent a promising avenue for further investigation of the potential applicability of exosomes in the development of vaccines against infectious diseases.

## Clinical Trials Using Exosome-Based Vaccines

Clinical trials using exosomes can be divided into three categories with different approaches. First, exosomes can be used as carriers to deliver drugs to specific targets. Second, exosomes derived from mesenchymal stem cells. And last, incorporating specific mRNAs and miRNAs into exosomes elicit responses in patients (179). In 2005, results from two phase I clinical trials using DEX vaccines were obtained. The first trial reported the use of DEXs loaded with HLA-restricted melanoma-associated antigen (MAGE) peptides, which were infused into patients with HLA A2+ non-small cell lung cancer (NSCLC) (41). After the administration of four weekly doses, the vaccine was well tolerated by all patients. However, only one-third of the patients presented with MAGE-specific T-cell responses, while two of the four analyzed patients showed an increase in NK cell activity (41). The second trial reported the use of DEXs derived from DCs pulsed with MAGE and inoculated them to conduct immunization of melanoma patients. No major toxicity event was reported by any patient, except for the occurrence of a grade I fever (five patients out of fifteen); however, no MAGE-specific response of CD4^+^ and CD8^+^ cells was observed in peripheral blood. Interestingly, NK cell effector functions were also induced by the DEX vaccine, where eight of the thirteen patients presented with an increased number of NK cells infiltrating the tumor site (40).

According to Fu and colleagues, the lack of an immune response to these vaccines can be associated with the DC type selected by researchers in these clinical trials (69). They used immature DCs, while other studies showed that exosomes derived from mature DCs induced more potent T-cell priming. A phase II clinical trial reported the use of DEXs derived from mature DCs pulsed with IFN-γ in patients with NSCLC, and no toxicity was observed, except for the occurrence of grade III hepatotoxicity in one patient. In this case, the DEX vaccine did not induce a cancer-specific T-cell immune response but resulted in the induction of NK cell functions (43). According to the authors, IFN-γ may lead to an upregulated expression of PD-1 ligands on DEXs, a well-known immune checkpoint that suppresses T-cell activity. Although these vaccines were designed to activate specific MHC-restricted T-cell responses, DEXs proved to be effective in activating NK cells in an MHC-independent manner. Interestingly, DEX-based vaccines have focused on direct CTL activation as an independent process in other immune cells. However, Näslund et al. showed that CD4^+^ T cells and B cells were necessary for the DEX activation of CTL anti-tumor response (85).

Recently, a non-randomized phase I/II clinical trial showed promising results with a vaccine designed using exosomes derived from DCs pulsed with SART1, a biomarker of squamous cell carcinoma of the esophagus. Pulsed DCs obtained from patients could generate exosomes that were well tolerated and induced antigen-specific CTLs in seven patients (180). One patient of this study remained stable for 20 months after DEXs therapy, although he developed lung metastasis after the stable period. The other six patients had progressive disease and died in a period up to 10 months after vaccination. These findings indicate that the development of a personalized exosome-based immunotherapy is feasible, although incredibly challenging. Patient indication criteria and the preparation of highly competent DCs for vaccine formulation are keystones of a successful exosome-based treatment (180). According to Xu and colleagues, it is important to investigate the anti-tumor immunity induced by DEXs-based vaccines to confirm whether DEXs can be used as tumor antigens for an exosome-based vaccine (52).

In addition to DEX vaccines, other clinical trials using different exosome-based vaccines have been reported. One phase I clinical trial reported the use of exosomes derived from ascites (AEXs) in combination with granulocyte-macrophage colony-stimulating factor (GM-CSF) as immunotherapy for colorectal cancer. Injection of AEXs for colorectal cancer was safe and well tolerated by all patients during the four weekly doses administered. Patients with advanced colorectal cancer subjected to treatment with AEXs plus GM-CSF demonstrated a strong anti-tumor cytotoxic T-lymphocyte response against the carcinoembryonic antigen (181), a colorectal cancer biomarker (182). Exosome-based vaccines have also been developed for the treatment of chronic diseases other than cancer. A phase II/III clinical trial was conducted using exosomes derived from umbilical cord MSCs in patients with chronic kidney diseases, such as type 1 diabetes and interstitial nephritis (45). The participants in the study reported no significant adverse effects during or after the treatment. The use of exosomes derived from MSCs improved overall kidney function and inflammatory immune activity. Currently, tests involving the safety and tolerance of aerosol inhalation of exosomes derived from MSCs are part of a clinical trial comprising healthy volunteers (NCT04313647). Another clinical trial involving he investigation of the use of exosomes derived from MSCs as a therapeutic strategy is underway against macular holes (NCT03437759).

### Clinical Trials Using Exosomes as a Potential Vaccine Against Coronavirus Disease (COVID-19)

More recently, due to the coronavirus pandemic, clinical trials for exosome-based therapy have shifted from cancer to COVID-19 treatment for future vaccine development (183). To this date, there are in total, 12 active clinical trials using exosome interventions at ClinicalTrials.gov. A phase I (NCT04747574) and a phase II (NCT04902183) independent clinical trials are recruiting patients with moderate or severe COVID-19 infection to evaluate the safety and efficacy of exosomes overexpressing CD24 of two doses with a patient follow-up for 23 days. CD24 is a costimulatory molecule expressed on several hematopoietic cells, especially progenitor cells, such as B cell progenitors (184). However, CD24 is also associated with autoimmune diseases (185, 186). Two phase I and II clinical trials are being conducted to investigate the safety and efficiency within 28 days after the first treatment of aerosol inhalation of bone marrow MSC-derived exosomes in severe patients hospitalized with SARS-CoV-2 pneumonia and COVID-19 (NCT04602442 and NCT04276987). And another phase I/II clinical trial (NCT04798716) is investigating the safety and efficiency of an intravenous infection of MSC-derived exosomes every other day on an escalating dose of 2:4:8 in the treatment of severe patients with COVID-19. According to these clinical trials description, MSC-derived exosomes may reduce lung inflammation and pathological impairment. Thus far, only one trial has reported results (NCT04491240), and no adverse events have been reported in patients after inhalation of 3 ml of MSC-derived exosomes twice a day for 10 days. However, there is no information about the source of MSCs used to generate exosomes and other relevant information concerning the aerosol formulation in this clinical trial. Additionally, another ongoing phase I/II clinical trial (NCT04389385) is investigating the safety and efficiency of inhaled exosomes derived from COVID-19 specific T cells that were activated and expanded *in vitro via* viral peptide exposure.

However, to this date, the clinical trials do not offer much information concerning the usage of exosomes to induce immunogenic properties and/or long-term memory response. The actual scenario of clinical trials using exosomes against COVID-19 is still evaluating safety and efficacy of exosome treatments. When completed, the ongoing clinical trials can provide the foundation for the conduction of future studies using MSC-derived exosomes in healthy patients. With their ability to elicit anti-inflammatory effects and modulate immune responses (187), MSC-derived exosomes may be important for the future design and development of COVID-19 vaccines.

Recently, a statement published by the ISEV and the International Society for Cell and Gene Therapy (ISCT) encouraged the conduction of further research and clinical trials using exosomes as a therapeutic strategy against COVID-19 (188). However, this statement also underscores the need for good clinical practice and rational clinical trial design.

## Conclusion

Initially, EVs were considered to demonstrate the sole function of cellular waste elimination; however, EVs are now recognized as crucial mediators of intercellular communication because of their capacity to deliver different molecules and transfer signals over long distances to modulate several physiological mechanisms. The immunomodulatory properties of EVs provide insights into their use as a cell-free therapeutic strategy for different diseases. Several studies have reported promising results on EV-based vaccines against different types of diseases, including cancer and numerous infectious diseases. However, exosomes from cancer cells modulate many aspects of intercellular communication, which they can play a crucial role in tumor progression and suppress anti-tumor activities. Understanding the dual effects of exosomes represent a major challenge for future therapies using exosome-based vaccines. Clinical trials showed modest results, with no antigen-specific response induced by exosome vaccines, i.e., MHC I/II-restricted TAAs did not stimulated anti-tumor properties in effector T cells. Further studies are needed to understand the pharmacokinetic of exosome-based vaccines. On the other hand, clinical trials revealed the ability of exosome-based vaccines in recruitment and activation of innate immunity. Further investigation is warranted for the development of new techniques for loading EVs with specific antigens or drugs, and for engineering EVs to display more efficiency in cargo delivery. When in combination with other therapies, exosome-based vaccines are more promising, for example, different studies showed that PD1/PDL1 blocking therapy combined with DEXs resulted in effective T cell activation (189). However, difficulties such as lack of quality control and standards for EV characterization and purification must be overcome. Also, logistical issues, such as manufacturing, storage, and administration of exosome-based vaccines need to be addressed (190). Additionally, exosome-based vaccination encompasses various issues on exosome biocompatibility for broad clinical usage and for the establishment of large-scale immunization programs. There are several challenges, including the development of an effective cell-free vaccine platform to use exosomes for the treatment of various diseases. A focus on such aspects and challenges is necessary for future exosome-based vaccine investigations.

## Author Contributions

FA conceived the idea. PS and FA designed, wrote, and edited the manuscript. All authors contributed to the article and approved the submitted version.

## Funding

This research was funded by Fundação de Amparo à Pesquisa do Estado de São Paulo (2019/25826-5 and 2016/03322-7); CNPq (Conselho Nacional de Desenvolvimento Científico e Tecnológico); CAPES (Coordenação de Aperfeiçoamento de Nível Superior); and Fundação de Apoio ao Ensino, Pesquisa e Assistência do Hospital das Clínicas da Faculdade de Medicina de Ribeirão Preto da Universidade de São Paulo.

## Conflict of Interest

The authors declare that the research was conducted in the absence of any commercial or financial relationships that could be construed as a potential conflict of interest.
